# Modelling human genetic disorders in *Xenopus tropicalis*

**DOI:** 10.1242/dmm.050754

**Published:** 2024-06-04

**Authors:** Helen Rankin Willsey, Eleanor G. Seaby, Annie Godwin, Sarah Ennis, Matthew Guille, Robert M. Grainger

**Affiliations:** ^1^ Department of Psychiatry and Behavioral Sciences, Weill Institute for Neurosciences, University of California San Francisco, San Francisco, CA 94158, USA; ^2^ Chan Zuckerberg Biohub - San Francisco, San Francisco, CA 94518, USA; ^3^ Genomic Informatics Group, Faculty of Medicine, University of Southampton, Southampton SO16 6YD, UK; ^4^ European Xenopus Resource Centre (EXRC), School of Biological Sciences, University of Portsmouth, Portsmouth PO1 2DY, UK; ^5^ Department of Biology, University of Virginia, Charlottesville, VA 22904, USA

**Keywords:** Disease, Genetics, CRISPR, *Xenopus tropicalis*

## Abstract

Recent progress in human disease genetics is leading to rapid advances in understanding pathobiological mechanisms. However, the sheer number of risk-conveying genetic variants being identified demands *in vivo* model systems that are amenable to functional analyses at scale. Here we provide a practical guide for using the diploid frog species *Xenopus tropicalis* to study many genes and variants to uncover conserved mechanisms of pathobiology relevant to human disease. We discuss key considerations in modelling human genetic disorders: genetic architecture, conservation, phenotyping strategy and rigour, as well as more complex topics, such as penetrance, expressivity, sex differences and current challenges in the field. As the patient-driven gene discovery field expands significantly, the cost-effective, rapid and higher throughput nature of *Xenopus* make it an essential member of the model organism armamentarium for understanding gene function in development and in relation to disease.

## Teaser

**Summary:** The diploid frog *Xenopus tropicalis* is a powerful model for unravelling conserved mechanisms underlying human genetic disorders. This Review discusses best practices for using these frogs to model disease.

## Introduction

Immense progress in exome- and genome-wide DNA analyses, coupled with the generation of large-scale genetic and phenotypic databases, has led to the curation of long lists of high-confidence disease-causing genes for a multitude of human genetic diseases affecting all tissues (Bamshad et al., 2019). However, the molecular, cellular, developmental and homeostatic functions of most of these genes are either unknown or poorly understood. Adding to this complexity, most genes are pleiotropic, so even when the function of a gene in one tissue at one developmental time is known, this knowledge may not translate to other tissues, times or contexts (Cummings et al., 2020; Panagiotakos and Pasca, 2022; Sestan and State, 2018; Willsey et al., 2018a, 2022b). This challenge of interpreting disease-risk gene lists can be readily met by using the high-throughput vertebrate model system *Xenopus tropicalis*, a diploid frog species (Bhattacharya et al., 2015; Blackburn and Miller, 2019; Deniz et al., 2018; Edwards et al., 2021; Exner and Willsey, 2021; Fainsod and Moody, 2022; Getwan and Lienkamp, 2017; Grainger, 2012; Griffin et al., 2020; Harland and Grainger, 2011; Hwang et al., 2019; Nakayama et al., 2015; Nenni et al., 2019; Willsey et al., 2022a). Like its closely related but allotetraploid species *Xenopus laevis*, *X. tropicalis* (hereafter also referred to as *Xenopus*) is amenable to fast genetic analysis of multiple organ systems simultaneously, affordably, and at scale (Abu-Daya and Godwin, 2023). Here we describe the unique advantages of the powerful model organism *X. tropicalis* for the diagnosis of human disorders and as a tool for understanding the molecular mechanisms underlying them. We also discuss the crucial considerations that are needed to promote rigorous and informative work when using *X. tropicalis*.

### Experimental advantages of *Xenopus* for modelling human genetic disorders

*X. tropicalis* has unique experimental advantages that render it well-suited for modelling human genetic disorders (Exner and Willsey, 2021). First, *X. tropicalis* has a diploid genome that is highly conserved between frogs and humans (Hellsten et al., 2010); its high level of synteny makes the identification of orthologous genes more straightforward than in animal models with less conservation and/or a duplicated genome. Second, the model organism database Xenbase (https://www.xenbase.org; RRID:SCR_003280) provides user-friendly access to the accurate, annotated reference genome, with excellent tools to facilitate genetic analysis and interpretation (Fisher et al., 2023; Fortriede et al., 2020). Additionally, methods for maintaining a thriving laboratory colony are well-established and costs significantly less than maintaining rodents (Nakayama and Grainger, 2023; Shaidani et al., 2020). *X. tropicalis* can be induced to mate year-round, with a single pair producing 4000+ embryos in a day by natural mating or by *in vitro* fertilisation (Lane and Khokha, 2022a,b). Embryos develop quickly; by day 4 they have developed organ systems, such as the central and peripheral nervous system, sensory organs, kidneys, skeletal muscle and cardiovascular systems, but not lungs or limbs. Within 10 days they exhibit robust quantitative behaviours that can serve as phenotypic readouts (Ismail et al., 2022).

Genetic perturbation protocols for *X. tropicalis*, including CRISPR/Cas9 mutagenesis (Bhattacharya et al., 2015; Nakayama et al., 2013), are well-established and cost-effective. Conveniently, mutagenesis of one of the two cells at 2-cell stage embryo yields a unilateral mutant, with one half of the animal carrying a homozygous mutation of interest while the other half serves as a within-animal control (DeLay et al., 2018; Macken et al., 2021; Willsey et al., 2018b). This is unique to *Xenopus* and makes generating thousands of mutant embryos per day feasible, thereby enabling truly parallelized analysis of dozens of risk genes by using a within-animal control. Indeed, this CRISPR-based strategy has a proven track record of exceptional success in studying autism spectrum disorders (ASD) risk genes (Willsey et al., 2021; Rosenthal et al., 2021), congenital anomalies (Kariminejad et al., 2019; Barbosa et al., 2020; Macken et al., 2021) cancer (Dimitrakopoulou et al., 2019), congenital heart disease (Deniz et al., 2018, 2023), kidney disease (DeLay et al., 2018; Getwan and Lienkamp, 2017) and many more (Edwards et al., 2021; Grand et al., 2023; Sega et al., 2019). Similarly, the developmental fate map of the early embryo has been described in detail (Nakamura and Kishiyama, 1971; Hirose and Jacobson, 1979; Jacobson and Hirose, 1981; Dale and Slack, 1987; Moody, 1987a,b; Masho, 1988; Masho and Kubota, 1986; Moody and Kline, 1990), allowing researchers to target genetic perturbations to specific tissues without using complex and time-consuming genetic methods.

Many transgenic and mutant lines are readily available, and there are protocols for generating new ones quickly (Aslan et al., 2017; Bhattacharya et al., 2015; Noble et al., 2022; Ogino et al., 2006; Shibata et al., 2022). Conveniently, The National Xenopus Resource (NXR; RRID: RRID:SCR_013731) and the European Xenopus Resource Centre (EXRC; RRID: SCR_007164) offer services for generating germline mutant lines for the community. Transgenic lines are proven tools for isolating tissues for single-cell analyses (Aztekin et al., 2019; Kakebeen et al., 2020), and protocols for isolating nuclei from mutant lines allow investigators to efficiently perform single-cell gene expression analyses comparing mutant and wild-type embryos (Nakayama et al., 2022a). The generation of mutant lines has recently been expedited by using gynogenesis protocols, in which the paternal genome is not inherited and researchers are, therefore, able to skip a generation in making homozygous mutants (Nakayama et al., 2022b). Similarly, a long history of genetic tool development and ease of phenotyping exists for *Xenopus*. Below, we expand on these and their most appropriate applications in this context. Another powerful feature of frog embryos and tadpoles is that they absorb small molecules from their surrounding culture medium, facilitating large-scale drug screening (Gull et al., 2023; Wheeler and Liu, 2012). In the search for targeted therapeutics for rare genetic disorders, this advantage is paramount.

Moreover, studying human disease genes in *Xenopus* has a history of producing phenotypes that often more closely recapitulate the human condition than even rodent models. For example, mutations in the essential eye transcription factor gene *pax6* in *Xenopus* result in a phenotype very similar to that of patients with the rare disease congenital aniridia (Nakayama et al., 2015), while mutations of this gene in mouse lead to a dissimilar ‘small-eye’ phenotype (Hill et al., 1991). In another example, genetic variants in the scaffold protein-encoding gene *USH1C* cause the rare disorder Usher syndrome 1C, and lead to both deafness and blindness in patients. Both the characteristic human eye and ear abnormalities are recapitulated in the *ush1c*-mutant of *X. tropicalis*, but the structure responsible for the eye deficit is absent in rodent photoreceptor cells and, thus, these animals are not optimal models for Usher syndrome 1C (Schietroma et al., 2017). The key point here is that mammalian models, despite their relative evolutionary proximity, do not always mimic all the features of a human disorder, and that non-mammalian models, like *Xenopus*, may offer advantages – either in phenotype as noted here or even when the *Xenopus* phenotype is not identical to that shown by humans. This is because the frog has advantages regarding the unique experimental strategies that are possible in this system. Finally, with respect to the responsible use of animals in research – i.e. the 3Rs: Replacement, Reduction and Refinement of animal experimentation, initially formulated by William Russell and Rex Burch in 1959 (see Hubrecht and Carter, 2019) – using *Xenopus* as a model rather than mammals is also attractive due to the absence of cerebral and limbic structures responsible for the translation of pain to distress, which are present in humans.

### Getting started – gene selection considerations

There are many routes to begin functional genetics work in *Xenopus*. For example, one group may begin with a single patient who presents with several potential disease-causing variants, and need to first prioritise variants for functional investigation by using *Xenopus*. Alternatively, another group may have a long-standing interest in a particular disease and want to model previously unseen variants observed in known disease-causing genes. In another scenario, a group may begin with an organ of interest and want to identify disease-causing genes that affect this organ. Finally, as the number of variants of uncertain significance (VUS) from DNA-sequencing projects continues to increase ever more rapidly, researchers may work in an integrated manner with clinical geneticists and bioinformaticians to test variant-disease links in *Xenopus*. The diverse array of starting points is a testament to the versatility of *Xenopus* functional genomics. Here we will focus on the first scenario, which highlights many of the core considerations that are paramount to any study modelling human disease genetics.

When considering multiple potential disease-causing variants within one patient, a helpful strategy for prioritising variants for modelling in *Xenopus* is to determine whether any loss-of-function (LOF) – i.e. frameshift, essential splice site or nonsense – variants in that gene ever occur in unaffected subjects. If LOF variants of a gene occur in unaffected controls, the gene in question is considered ‘LOF tolerant’ and not to be under evolutionary constraint, so is less likely to be disease causing. To quantify LOF constraint, there is a metric called the LOF observed/expected upper-bound fraction (LOEUF) score (Karczewski et al., 2020). Genes with a low LOEUF score are intolerant to gene perturbation and, thus, provide clues to biological essentiality (Karczewski et al., 2020). Indeed, genes with the lowest LOEUF scores are those most enriched for known disease genes and represent targets for novel disease gene associations (Seaby et al., 2022). Therefore, LOF constraint can help prioritise which genes to study in model systems. LOEUF scores can be found by searching the Genome Aggregation Database (gnomAD), which to date represents the largest population dataset globally from more than 195,000 individuals, curated from over 60 studies (https://gnomad.broadinstitute.org/) (Gudmundsson et al., 2022; Karczewski et al., 2020) (Table 1). Importantly, this database is generated from unaffected individuals and, therefore, rare paediatric-onset diseases are underrepresented in it, meaning that monogenic disease-causing variants, many of which will be of interest to *Xenopus* researchers, are largely absent from this database. Rather, this database is a helpful resource for ascertaining the baseline variation that occurs in a gene of interest in an unaffected population. An abundance of LOF variants within a gene of interest in an unaffected population argues against its relevance to disease.

**Table 1.**
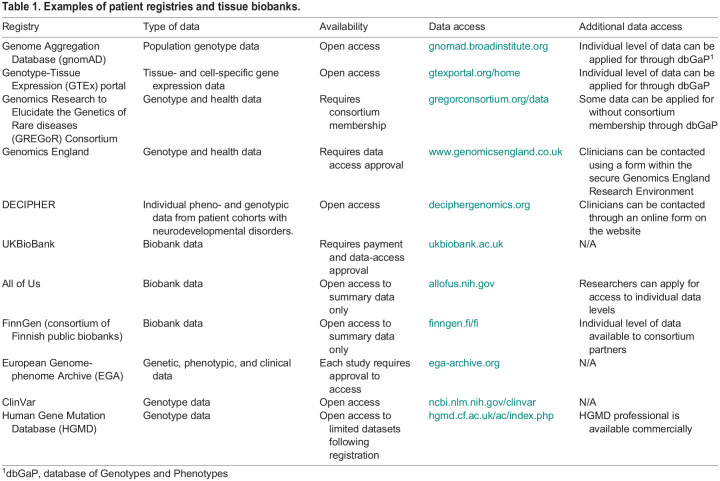
Examples of patient registries and tissue biobanks.

Another consideration when prioritising potentially pathogenic genetic variants for modelling is whether these have already been noticed in patient populations. Databases where this can be explored online exist (Table 1), and they often include the genomic location, gene/protein variant, higher level phenotyping terms, clinical significance and, occasionally, additional incidental findings. One of the most-recognised genetic disease databases is the Online Mendelian Inheritance in Man (OMIM) database (https://www.omim.org/), a manually curated open-source website that describes human genes, genetic disorders, inheritance patterns and genotype/phenotype relationships (Table 1) (Hamosh et al., 2005). Another widely used human disease dataset is the DECIPHER (https://www.deciphergenomics.org/) online repository of genetic variation with associated phenotypes for >45,000 patients with rare disorders (Table 1) (Firth et al., 2009). The freely available DECIPHER browser enables the user to search genes, regions and phenotypes for patients with matching variants. These databases can also be especially helpful if an investigator is starting with a disorder of interest and looking for disease-causing genes to study. To preserve patient anonymity, only high-level phenotypes (e.g. abnormality of the cardiovascular system, etc.) are displayed. However, if more detailed clinical information is required, one can contact the clinician or principal investigator of the participating centre where that patient was recruited. This can facilitate access to valuable additional clinical information, such as when the patient was last assessed, updates on disease progression and access to information on co-morbidities or additional damaging variants.

Another very helpful database is ClinVar (https://www.ncbi.nlm.nih.gov/clinvar; Landrum et al., 2014), an open-access repository for the standardised classification of variants (Table 1) (Landrum et al., 2014). Anyone using the platform can submit a variant found in a patient sample and assert its clinical significance by applying a standardised set of guidelines used for diagnostic reporting as set out by the American College of Medical Genetics (ACMG) and/or provide supporting data (Richards et al., 2015). Therefore, the database can be queried for variants previously described and validated as pathogenic by ACMG guidelines. ClinVar benefits from an easy-to-use graphical interface enabling researchers to see where the different classes of variants lie across the gene, and links to dbVar, which holds chromosomal information, and to LitVarz, which allows interrogation of the literature based on specific variants and genes.

Once a potentially disease-causing gene and/or variant has been identified, an important strategy when deciding whether they could be productively modelled in *Xenopus* is to align the protein sequences from *X. tropicalis* and human by using the NCBI tool HomoloGene (https://www.ncbi.nlm.nih.gov/homologene). This can help determine conservation at protein level – which, in turn, may help selecting reagents, such as antibodies – and whether generating mutant lines with patient-derived variants is possible. Xenbase should also be consulted to assess genomic synteny and to determine which isoforms have been described in *Xenopus*, and when and where in development the gene is expressed (Bowes et al., 2010; Fortriede et al., 2020). This early effort can also preempt any potential issues with genome annotation. Finally, other databases, e.g. ModelMatcher (https://www.modelmatcher.net/) and those specific for model organisms, should be checked for existing but unpublished models of the disease gene, either to inform experimental design or to prevent unintentional duplication of effort.

### Designing strategies that reflect the genetic architecture of the disorder

Once a gene(s) of interest has been selected, the next major consideration is the nature of the patient alleles and the genetic nature of the disorder of interest. For example, one would model a single-gene recessive disorder (Appendix G, Single-Gene Disorders, 2010), such as cystic fibrosis or phenylketonuria, very differently from a complex heterogenous genetic disorder, like autism, or a disorder in which the variants are gain-of-function (GoF), like Noonan syndrome (Roberts et al., 2007). Therefore, familiarity with common genetics terminology and concepts is essential. Here we briefly explore the most critical ones encountered in patient variant modelling.

**Fig. 1.**
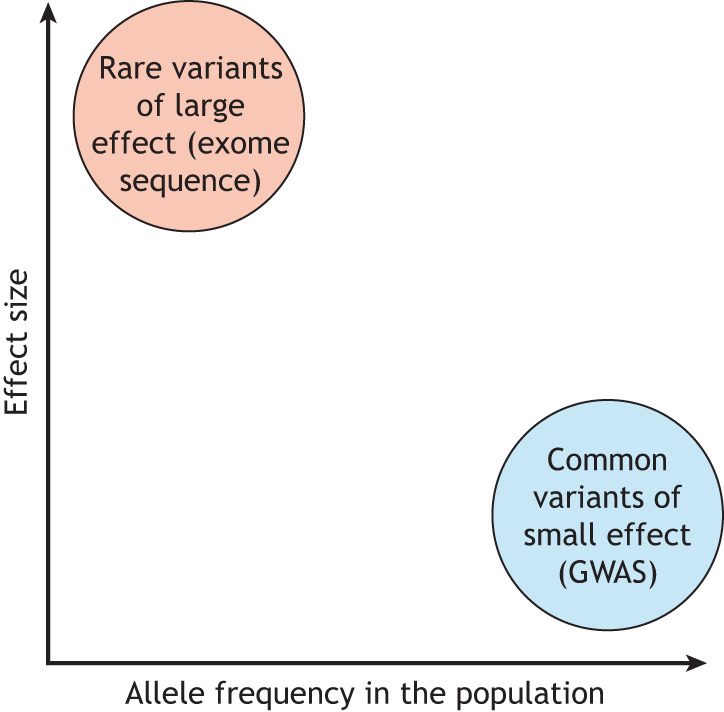
**Relationship between rare and common variants with respect to allele frequency and effect size.** Rare variants are typically detected in specific, clinically indicated patient trio (i.e. involving testing of the affected individual and both biological parents) studies of exome sequencing, and enriched for *de novo* variants with individually large effects. When perturbed, the genes that carry these variants often produce phenotypes in model organisms. By contrast, common variants are usually identified in much broader genome-wide association studies (GWAS) of large unrelated cohorts. Individually, these common variants carry very small risk and are unlikely to produce a measurable phenotype when recapitulated in model organisms.

One essential concept is that of variant effect size and it is critical for modelling genetic disorders in *Xenopus*. Common variants, where population allele frequency exceeds 1%, as identified in genome-wide association studies (GWAS), carry very small individual effect sizes. Any individual variant only increases risk very slightly (Fig. 1) but, because they are common, they carry population-level risk. By contrast, rare variants with population allele frequencies of <1%, as typically identified in patient trio exome sequencing, carry large individual risks (Fig. 1). This way, it is much more likely to observe a phenotype in *Xenopus* when recapitulating rare variants from patient sequencing data than common ones. Common variants, often referred to as single nucleotide polymorphisms (SNPs), are also often in intergenic regions and it can be difficult to reliably assign the affected gene (reviewed by Boyling et al. 2022). Rare variants from patient exome sequencing studies are enriched for protein-coding variants that often directly alter protein structure, while common variants often reside in non-coding regions that can be more difficult to interpret (reviewed by Allou and Mundlos, 2023). This makes patient-derived rare variants more straightforward to model and generally more suited to model organism studies, particularly when LOF variants in the same gene are reported in multiple unrelated affected individuals, since this strengthens the likelihood that variants in that gene are pathogenic.

Copy-number variants (CNVs) often have intermediate effect sizes and allele frequencies (Willsey et al., 2022b), and can be modelled in *Xenopus*, with some caveats. These variants often span large regions of the human genome, so assessing synteny and conservation between *Xenopus* and human is paramount. This is particularly important as intergenic regions usually do have larger evolutionary differences than coding regions. Nevertheless, to model CNVs with CRISPR, one can design two single guide RNAs (gRNAs) that flank the mapped orthologous CNV breakpoints and generate large deletions. One can also inject larger DNA constructs to model duplications. *Xenopus* embryos will tolerate exogenous DNA fairly well, though the inheritance of the construct can be rather mosaic (Cleaver and Krieg, 1999), so it is recommended to include a visible marker within the construct to track expression patterns, or to breed the animals, which is time consuming.

Where LOF variants are implicated in disorder pathobiology, simply perturbing a single gene is the simplest way to model using CRISPR-based genome editing. One can design an sgRNA that directs Cas9 to cut near the 5′ end of the coding sequence to empirically test editing efficiency, and choose sgRNAs of high efficiency and specificity, with the assumption that the cuts will be repaired by non-homologous end-joining, thus, most likely generating deletions and, therefore, frameshift mutations that will result in a non-functional gene product (Willsey et al., 2022a). Unlike LOF variants, the impact of missense variants can be harder to interpret, although there are algorithms that will predict how deleterious a given variant would be to protein structure and, therefore, function. Missense badness, PolyPhen-2 and missense Constraint (collectively forming the MPC) score are some examples of these algorithms (Samocha et al., 2017 preprint). Mimicking missense variants will have a very high impact on the field, since a large proportion of disease variants are missense by nature. Base editing, which changes a single base without cutting the DNA backbone and is, therefore, perfectly suited to missense modelling (Komor et al., 2016), has been shown to work in *Xenopus* (Shi et al., 2019; Park et al., 2017; Naert et al., 2020 and 2021b). *Xenopus* are particularly suited for this type of modelling due to the ease of generating many mutant lines. There is also the potential for doing this work in the generation F_0_, given the high tolerance of injected ribonucleoprotein complexes in the frog.

When modelling a complex genetic disorder where many different genes carry risk, such as most psychiatric disorders, including autism and schizophrenia, there are advantages to working in *Xenopus*. In this case, most of the genes involved are highly pleiotropic, and it is difficult to separate the pleiotropy of a gene from the relevant function in the disorder just by studying individual genes (Willsey et al., 2022b). Instead, it is straightforward in *X. tropicalis* to study many genes and/or variants in parallel because of the speed, ease and cost-effectiveness of CRISPR mutagenesis. In one afternoon, thousands of mutant embryos can be created for many different genes. This can allow for the identification of gene functions and phenotypes that are common for different genes associated with a particular disease of unknown pathobiology. For example, CRISPR perturbations of any of the ten genes with the strongest statistical evidence for association with autism did, individually, cause neurogenesis defects in *Xenopus*, highlighting this process as one potentially relevant to the disorder (Willsey et al., 2021). This ‘phenotypic convergence’ can also combat the formidable problem of pleiotropy, as studying just one gene at a time may lead to focusing on a phenotype specific to that particular gene and not phenotypes more broadly relevant to other disease-risk genes (Willsey et al., 2021, 2022b). Similarly, performing *in situ* hybridizations (Willsey, 2021) or screening single-cell transcriptomic atlases (Briggs et al., 2018) for many risk genes in parallel can help limit the search space for phenotypes by identifying tissues, cell types and timepoints of co-expression.

### Building gene-specific tools

**Fig. 2.**
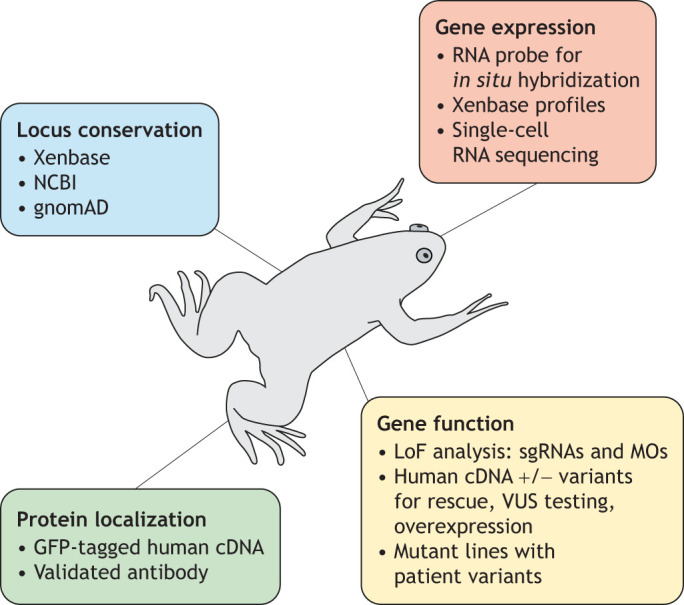
**Approaches and tools that facilitate human genetic disease modelling in *Xenopus*.** Once a gene of interest is selected, the locus conservation between *X. tropicalis* and humans can be explored in Xenbase, NCBI and gnomAD databases. The expression profile of the gene can be queried by using RNA *in situ* hybridization and on Xenbase. Then the protein can be located in cells and embryos through expression of a tagged human cDNA clone or validated antibody staining. Finally, the function of this gene can be inferred through loss-of-function (LoF) analysis, rescue experiments, overexpression and generating mutant lines with patient-derived variants. This kind of work can quickly point to mechanisms of disease, and tissues and structures for successful studies.

Once a gene is selected for investigation, and direction of perturbation and expected effect size have been considered, it is advantageous to begin developing gene-specific tools (Fig. 2). Often, it can be extraordinarily helpful to determine the developmental expression patterns for a gene, as this may point to relevant tissues affected by genetic perturbation and its function. For example, exclusive expression in highly ciliated tissues may suggest a potential function in ciliary biology and, consequently, a role in ciliopathies. This is particularly useful when studying syndromic conditions that affect multiple tissues and require an RNA *in situ* hybridization probe which, in turn, require a DNA template. *Xenopus* plasmid libraries of such constructs exist, such as the ORFeome (Grant et al., 2015) and the I.M.A.G.E. database (Lennon et al., 1996). Many *Xenopus*-specific constructs, including those donated from laboratory collections, can currently be obtained from horizon (https://horizondiscovery.com/en) or the EXRC. If unavailable, plasmids can be generated by PCR from a cDNA library and simple cloning, which is particularly straightforward in *Xenopus* because of the high-quality accessible genome sequence and the ease of which large amounts of stage-specific cDNA can be obtained. It is important to remember that the composition of RNA isoforms may change during development and/or by cell type, and it is advisable to select or engineer the probe that matches the timepoints and tissues of interest, or the one that recognizes a coding sequence that is present in all isoforms.

For studies that require probing the product of the gene in question, investing in a reliable antibody against the *Xenopus* protein of interest can be exceptionally helpful for validating reagents, such as sgRNAs, by confirming that protein levels are altered, and for localising the protein in cells and tissues. However, all antibodies must be rigorously validated for specificity and avidity, ideally through confirming the antibody signal using immunohistochemistry or western blotting, in tissues that express the target and seeing it abolished in mutant tissues where the target is absent.

For LOF analyses, developing multiple orthogonal perturbation reagents is advisable. For CRISPR-based gene knockouts, sgRNAs can be easily designed through online algorithms (Moreno-Mateos et al., 2015; Labun et al., 2019). We also recommend that multiple non-overlapping target sequences are used, as discussed below in the rigour section. While these algorithms will predict cutting efficiencies, the actual efficiency for each sgRNA must be empirically determined in *Xenopus* embryos. One strategy is to use Sanger sequencing of injected animals followed by sequence-trace deconvolution, and estimation of the insertion and deletion frequencies (Brinkman et al., 2014; Conant et al., 2022). Strategies also exist to maximise phenotypic penetrance of any gene edits in generation F_0_ to avoid in-frame mutations (Naert et al., 2020). A helpful orthogonal strategy to CRISPR-based gene knockout is to aim for post-transcriptional knockdown of the target. For this, the first step is to generate and validate a morpholino oligonucleotide, targeting either the start codon – thereby, blocking translation of mRNA, or a splice site – thereby, blocking RNA splicing (Malartre et al., 2006). Morpholinos can be helpful by strongly knocking down expression of the target gene, targeting specific isoforms or depleting maternal RNAs from the zygote. The latter is particularly useful for studying the LOF of maternally expressed mRNAs, since CRISPRs will only target the zygotic genome.

Finally, if the protein of interest is amenable to pharmacological modulation, e.g. by using an inhibitor to block a kinase, this can be a helpful additional path for orthogonal perturbation. Pharmacological inhibitors have the additional advantage of temporal control, as the drug can be added to the embryo culture medium at different times during development, separating pleiotropic roles or bypassing early lethality. Ideally, the targeting specificities of such reagents are controlled by using multiple compounds to target the same protein. For CRISPR experiments, another possible control is to target any potential off-target sites revealed by the algorithm used to choose the sgRNA, to ensure one does not observe the on-target phenotype.

Given the need for relevance to humans, modelling disease-causing variants extends beyond LOF. Expressing the human coding sequence can be very helpful for GoF, localization and rescue experiments, and/or for patient variant testing. It should be noted that transgenic expression of human genes risks disrupting protein-protein interactions by crossing species if these interactions are not evolutionarily conserved, as well as issues with inappropriate stoichiometries and potential toxicity due to high dosage.

### Designing phenotyping assays

Once a disease-causing gene has been selected and the perturbation strategy optimised, developing a phenotyping pipeline for *Xenopus* is straightforward since many tools have been developed and used for embryology studies in the last 60 years. Fortunately, robust transcriptomic, *in situ* hybridization and proteomics data are available on Xenbase to guide the focus of phenotyping assays. Referring to transcriptomic and proteomic data can also inform whether any maternal proteins persisting in the early embryo may mitigate phenotype presentation in early development. During phenotypic experiment design, it is useful to plan and conduct pilot studies to help estimate sample sizes, and to investigate the extent of variability in the phenotype – particularly when analysing mosaic generation F_0_ models. The first readout after a genetic perturbation is usually brightfield microscopy of the whole animal to identify gross changes and their relationship to regions of expression or clinical presentation in humans. Aside from being broadly accessible in every laboratory, whole-animal examination is important since disruption of gene function in distinct tissues and at different stages within the whole organism may generate complex phenotype alterations.

**Fig. 3.**
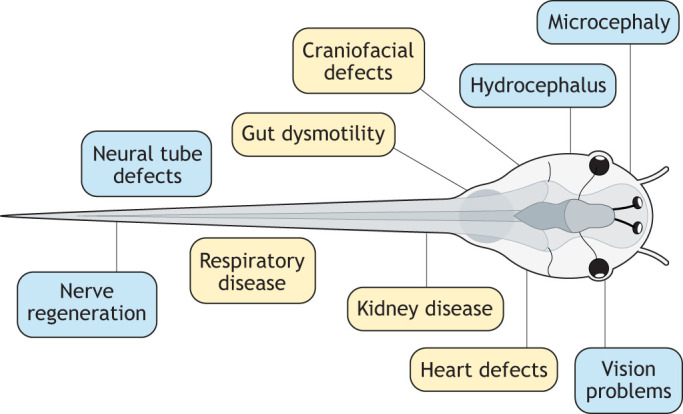
**Phenotyping opportunities in *Xenopus*.** Studying how genetic perturbation disrupts organ function is essential to successful human disease modelling. Various methods are available in *Xenopus* and, due to the functional similarities, can be applied to characterising disorders within organs as diverse as lung, heart, brain, kidney and gut.

Once the affected organ(s) or cell type(s) have been identified, key tissue-level and molecular differences can be assessed in more detail in transgenic *Xenopus* lines or with techniques, such as *in situ* hybridisation and immunohistochemistry. For most human organs an orthologous *Xenopus* organ exists at tadpole stage (∼6 days after fertilisation), and these organs can be studied by simple histological staining and microscopy techniques (Fig. 3). More subtle morphological changes can be captured by state-of-the-art imaging techniques, including MicroCT, light sheet and optical coherence tomography (Date et al., 2019; Deniz et al., 2017; Macken et al., 2021; Naert et al., 2021a,b). Detailed image analysis capacity is being significantly improved by machine-learning algorithms capable of detecting differences between control and mutant samples (Naert et al., 2021a,b).

As advanced research elucidates the nature of more rare human genetic diseases, modelling studies will need to adopt additional sophisticated methods of phenotyping. For example, in response to the large number of neurodevelopmental disorders identified by diagnostic sequencing and analysis, quantitative behavioural assays are now being applied to *Xenopus* disease models. At present, these include analysis of locomotion and working memory (Ismail et al., 2022) as well as epilepsy (Panthi et al., 2023), and mainly use established video-imaging methods and analysis algorithms adapted for *Xenopus* (Ismail et al., 2022; Panthi et al., 2023). These assays have the potential to generate high-throughput data with which to study neurodevelopmental disorders and test repurposed licenced medications (see e.g. the target-agnostic drug discovery platform CogniXense, https://wyss.harvard.edu/technology/cognixense-for-rare-disease-drug-discovery/). Work of this nature is being complemented by the historic strengths of the *Xenopus* model, e.g. by using techniques to record neuronal plasticity and action potentials both within intact tadpoles, and in primary cultures of neuronal cells or neuronal/muscle co-cultures derived from embryos with gene variants (Sun and Poo, 1985; Gore et al., 2021; Hiramoto and Cline, 2021).

### Rigour

As discussed previously, CRISPR/Cas9-based editing has become a popular approach for the perturbation of gene function when modelling human genetic disease in *Xenopus.* For these experiments, the recommended controls vary greatly, from using non-coding or scrambled sgRNAs to the use of sgRNAs targeting positive-control genes, like pigmentation genes or disease genes in other tissues (Willsey et al., 2021). In addition, investigators also often use multiple sgRNAs targeted to the same gene, as we recommended above, and test whether the edits induced by individual sgRNAs phenocopy each other (Willsey et al., 2018b). The ultimate control, of course, is rescuing the phenotype by expressing the reference allele (Willsey et al., 2020). This rescue approach also opens up the huge opportunity to test VUS and/or missense variants to infer causality of suspected alleles (Blackburn et al., 2019; Kostiuk and Khokha, 2021). The sequence of the expression construct can be modified with said variants, and if this modified allele is not able to rescue the phenotype, like the reference allele does, this indicates that the missense variant or VUS causes LOF. The rescue allele is most often cloned into an expression plasmid to make synthetic mRNA and is then microinjected. Other vectors include a bacterial artificial chromosome construct (Fish et al., 2014) or knock-in of the desired template into the genome (Nakayama et al., 2020), although these strategies can be technically challenging. Regardless of the rescue allele strategy, a lack of phenotypic rescue does not necessarily mean that the original perturbation was off-target or non-specific. There are many reasons why a rescue experiment may not work, including inadequate gene dosage, cell-type-specific gene expression or temporal dynamics.

Additional experimental controls include genetic perturbation of one cell of a 2-cell-stage embryo of generation F_0_, which allows within-animal comparisons and controls for inter-animal variation, especially when the perturbation is coupled with the expression of a fluorophore for tracing. Phenocopying a LOF perturbation with an additional technique, such as a pharmacological inhibitor or expression of a dominant-negative construct can increase confidence that the observed phenotype is caused by LOF of the gene in question. Generating a germline mutant line that recapitulates the F_0_ phenotype can be very powerful confirmation, albeit much more time consuming. While more challenging experimentally, creating a germline benign variant in the gene of interest that does not produce the phenotype in question can be powerful evidence that mutagenesis itself did not create the phenotype. Also, whenever possible, the identity of control and test animals should be masked to the researcher performing the phenotyping to prevent bias. Finally, we advise being cautious when including detailed clinical phenotyping into the design phase of these experiments, since this can bias findings and may also lead to overlooking phenotypes in the frog that are either species-specific or have yet to be identified in the often very small number of human patients with a particular gene variant.

### *Xenopus* as a diagnostic platform

Whilst the ability to sequence DNA has advanced tremendously, the ability to interpret variants significantly lags behind. At present, ∼70% of variants captured in the sequencing results of a patient are VUS, which may be in known or newly identified disease-causing genes. VUS are usually coding variants that lack sufficient evidence to determine whether they are pathogenic or benign. Whilst we briefly mentioned expressing VUS in *Xenopus* in the previous section, it is important to emphasise that testing the link between a VUS and disease is significantly different from the LOF studies described above. When attempting to model a VUS in a gene that has not been previously associated with a disease, a simple LOF strategy as outlined in detail above could be a useful first step, although LOF models are insufficient to underpin clinical intervention. However, if a patient-specific VUS is linked to a known disease-causing gene, only precise models of the variant have merit. Even when a novel variant is identified in a gene that, when inactivated, is entirely consistent with the phenotype of a patient, said variant will still be classified as a VUS and often not clinically reported unless functionally validated. Therefore, there is strong demand from genetic disease patients, families and clinicians for cost-effective and rapid *in vivo* models to functionally test these variants and improve diagnostic rates.

*Xenopus* provides an excellent model for this scenario, offering multiple possible approaches. First, the VUS can be recapitulated in the endogenous *Xenopus* gene if this is sufficiently conserved. Whilst this is straightforward for LOF frameshift, deletion, exon skipping and nonsense mutations, single-base changes, however, are more of a challenge and require the improvement of base-editing techniques in the frog (Shi et al., 2019; Park et al., 2017; Naert et al., 2021a,b). Heterozygous, non-mosaic, precise changes, including small insertions, have been achieved by engineering the oocyte genome (Aslan et al., 2017; Martin, 2023) or by injecting long single-stranded DNA templates for insertion into the frog genome via endogenous homology-directed repair (Nakayama et al., 2020, 2022c).These methodologies remain inefficient yet worthwhile.

An alternative approach that mitigates the inefficiencies of engineering precise gene edits is to first disrupt the endogenous *Xenopus* gene and then attempt to rescue its expression, as we discussed above. This can be achieved using a human cDNA expression construct that either carries the reference sequence or one with the VUS (Devotta et al., 2016; Griffin et al., 2018). If the reference sequence can rescue the phenotype but the VUS cannot, that provides excellent evidence that the VUS is deleterious to protein function. The largest issue with this approach is that, in some cases, the human reference sequence cannot rescue the frog phenotype for a variety of possible reasons, including dose, failure to interact properly with *Xenopus* proteins and cell type specificity (discussed by Rana et al., 2006). Alternatively, if the VUS but not the reference allele produces a LOF phenotype, this can be evidence of a dominant-negative mechanism of genetic perturbation. Finally, but perhaps less convincingly, overexpression of the reference sequence can be used to determine the GoF outcome and then this phenotype can be compared with that produced by expression of variants.

In terms of controls for diagnostic work, if the VUS in frog recapitulates the phenotype of the patient, then this already shows the result is consistent in two different species. Coupled with a second experiment that confirms the phenotype in a biochemical or cellular assay, two species sharing a VUS-phenotype link is strong evidence for specificity and relevance in experiments testing the link between a genetic variant and disease. Of course, there is also the potential for the frog to incompletely recapitulate the human presentation due to evolutionary differences. In this case, a complementary model, perhaps human iPSCs or a mammalian model, can be useful to fill in those gaps.

In addition to VUS classifications, *Xenopus* is an excellent platform for modelling diseases with known causal genetic variants but unknown molecular mechanisms of disease (Mascibroda et al., 2022; Wong et al., 2023; Marquez et al., 2021; Colleluori and Khokha, 2023; Sempou et al., 2022; Willsey et al., 2021; Rosenthal et al., 2021; Grand et al., 2023; Getwan et al., 2021; Kulkarni and Khokha, 2018; Boskovski et al., 2013). In this case, specific variants can be studied, and molecular mechanisms identified *in vivo*. As many disease-causing variants are missense and, potentially, not simply LOF, *Xenopus* is extremely valuable because it is cost-effective and simple to create many germline mutant lines to mimic such variants in their native locus, as long as the locus is well-conserved between humans and frogs. Such work identifying new disease mechanisms can open the door to biomarker and therapeutic development.

### Working with clinicians and ethical considerations

When attempting to model VUS and potential new disease gene(s), there are clear benefits to having direct contact with the primary referring clinician or bioinformatician. Through direct collaborations with physicians and clinical geneticists, researchers can ascertain the variant-calling methodology and the confidence in the *in silico* predictions of pathogenicity for each variant (Seaby and Ennis, 2020). One challenge associated with this collaboration is that patient genetic and phenotypic data are subject to extensive protections to ensure privacy. Whilst medical privacy regulations vary between countries, most databases generally restrict access to data that may potentially identify the patient. For example, when mining rare disease data from the Genomics England database, it is not possible to export any human phenotype ontology (HPO) terms (Robinson et al., 2008) or a combination of HPO terms that occurs in fewer than five individuals, regardless of genotype data. In the USA, access to clinical data for research purposes often requires Institutional Review Board approval and a data transfer agreement. Such restrictions, even for de-identified and/or fully anonymised data, frequently vary between repositories (Table 1) and researchers need to learn to navigate them prior to planning to model the variants in *Xenopus* and other organisms.

An additional consideration is that there are significant differences in the way that *Xenopus* researchers and clinical geneticists describe changes to the genome. A small, but crucial difference that has reduced cross-disciplinary fluency in the past is that clinical or human geneticists consider the first nucleotide of the translation start codon in the reference sequence as nucleotide 1 of a coding DNA (den Dunnen et al., 2016), while animal researchers normally use the start of the transcript in the annotated genome as nucleotide 1. Similarly, many datasets and clinical reports do not include information on the zygosity of the variant. The short but excellent guide on how variants are annotated in human genetics is thus essential reading (Ogino et al., 2007). Less of a systematic challenge is that the workload of clinicians may not give them time to record the complete phenotypic information about a patient. For this reason, it is often desirable to work with clinicians who are based locally to the model organism laboratory and, ideally, with whose team researchers have close and regular collaboration. This allows researchers to follow up on specific, additional phenotypes that may present in the model but have not been recorded in the patient reports. Comprehensive phenotyping information is often very difficult to find in publicly accessible databases. By working closely with bioinformaticians and clinical geneticists, *Xenopus* researchers can not only enhance both their own understanding of the phenotypes and genotypes associated with each disease, but can also have rapid and powerful impact by testing genotype-phenotype links and helping clinicians support their patients and their families.

Accessing information through regulatory bodies and patient advocacy groups is an important approach, but involves a significant investment to ensure ethics and informed consent are considered properly. However, including these organisations is valuable since they offer opportunities to access patient registries, tissue biobanks and global collaborations, which improves the depth of data available. In particular, these groups often facilitate contacts with those working in different models, whether cells, organoids or other animals. It is increasingly recognised that multi-species comparisons are important for improving our understanding of gene-phenotype relationships and the key molecular findings linked to the perturbation of gene function in the context of human disease modelling and beyond.

To mitigate and anticipate the above issues in data sharing, patient consent, and ethics, the community is developing new data-sharing models. A prime example is the Centers for Mendelian Genomics (CMG) in the USA, now governed by the GREGoR Consortium (Bamshad et al., 2012). At recruitment for GREGoR, patients are consented for sequencing and for anonymised sharing of their phenotype and genotype data globally with other clinicians and researchers working on the same gene/disease. In GREGoR, an investigator will usually recruit patients locally and patient DNA will be sequenced by one of the CMG sites. Sequenced and processed data are stored on a secure, central cloud solution, keeping one copy of the data in a central repository (Schatz et al., 2022). The CMG analyses the cloud-based genomic data with the investigator and makes it accessible for further study. For example, data from a Swedish cardiovascular disease cohort may be pooled and analysed together with that of a similar cohort from Ecuador. All CMG data are added to the database of Genotypes and Phenotypes (dbGaP) (Tryka et al., 2014) so that external researchers may apply for access. Since each CMG cohort has a named principal investigator, there is a clear line of communication for external researchers to liaise with investigators and clinicians, particularly to address demand for additional clinical information and for new samples for future research. Whilst the use of patient data is heavily regulated in some countries and this adds administrative requirements, it is important to recognise that using patient data from other countries for disease modelling in *Xenopus* may be unethical, depending on the nature of the permissions obtained.

### Potential complications when working in *Xenopus*

There are obvious differences between *Xenopus* and humans which must be considered when designing human disease modelling experiments and interpreting phenotype data. These include neuroanatomical differences, the development of pronephric kidneys, a three-chambered cardiac structure, the process of metamorphosis, development of septated lungs, storage of fat in fat bodies and the process of skeletal development (Carotenuto et al., 2023). Not all differences should, however, be considered limitations. Despite having only one heart ventricle, *Xenopus* have atrioventricular valves, ventricular trabeculae, asymmetric atrium, and a similar *in vivo* cardiac orientation with left curvature of the outflow tract, which are sufficient anatomical structures to allow modelling of many cardiac abnormalities. Additionally, *Xenopus* tadpoles survive for an extended period with minimal cardiovascular function, enabling researchers to study end-stage cardiac deterioration (Smith et al., 2020) (reviewed by Hoppler and Conlon, 2020). There are many examples of key mechanistic data with human relevance being discovered in *Xenopus* organs that differ from humans (reviewed by Blackburn and Miller, 2019; Exner and Willsey, 2021; Womble et al., 2016).

Whilst *Xenopus* development is beautifully described and understood (Zahn et al., 2022), the focus of research has historically been on pre-metamorphic stages. When modelling human disease, it can be advantageous to directly compare the disease course, physiology, development and ageing processes of the model organisms to that of humans. Although there are many examples of such comparative (patho)physiology in the mouse (reviewed by Cacheiro et al., 2020; Potter et al., 2016), these broader longitudinal studies are currently lacking in *Xenopus* and an aged *Xenopus* model seems difficult to generate due to their prolonged lifespan, which commonly exceeds 15 years in the laboratory.

Bridging between humans and *Xenopus* will invariably raise classic questions of penetrance and expressivity. In human populations, disease-linked variant penetrance and expressivity often varies significantly between individuals (see e.g. Solomon et al., 2009; Mecklenburg et al., 2021). Similarly, human disease-associated variants may present somewhat differently than null variants do in mice or *Xenopus*. This could reflect evolutionary differences in genetic background or that the human variants are not null variants and, instead, could be productively modelled as germline mutant lines in *X. tropicalis*, if the locus is well conserved.

Finally, an important variable to consider when modelling human genetic disorders is the effect of biological sex. Many genetic disorders are sex biased, especially psychiatric disorders, with important implications and opportunities for modelling (Christiansen et al., 2022). *Xenopus* have a genetic mechanism of sex determination. Although the sex determining-gene is currently unidentified in *X. tropicalis*, it is well characterised in the closely related species *X. laevis* (Yoshimoto et al., 2008). Conveniently, frog gonads form later than most other organs, morphologically and molecularly diverging at the beginning of metamorphosis (Piprek et al., 2017). As the majority of disease modelling work in the field right now uses frogs at earlier developmental stages, the effect of gonadal biology on disease course is bypassed. Convenient experimental manipulations can, however, affect sex determination: treating animals with estradiol causes sex reversal in genetic males and aromatase inhibitor treatment reverses the sex of genetic females (Chang and Witschi, 1956; Olmstead et al., 2009; Villalpando and Merchant-Larios, 1990). These tractable approaches may be useful for understanding the influence of biological sex on specific diseases in the future. Overall, *Xenopus* is a powerful model for diagnosing and modelling rare diseases, but there are challenges that researchers will often need to address.

### Outlook

As our knowledge of human disease genetics expands, *Xenopus* is available to meet the fast-growing need to model gene and variant function *in vivo*. With a wide variety of experimental techniques and opportunities for many organ systems at scale, *Xenopus* will be a key model for working quickly and affordably. The ability to study many genes and variants in parallel in a diploid vertebrate is an unparalleled advantage of the system, and provides the opportunity to combat genetic pleiotropy and meet the challenges of modelling complex genetic disorders. Further, *Xenopus* holds great promise for studying particular variants of interest since it is feasible to generate and study many germline mutant lines in parallel. This is especially attractive as many human disease-causing variants are often not simply LOF. This approach maintains the appropriate genomic context of the variant and allows for the dissection of the precise effect of the variant. Finally, given the ease of drug screening, *Xenopus* can also provide a simple *in vivo* therapeutics screening platform.

Currently, a main barrier to success is a disconnect between clinicians, geneticists and model organism experts (Khokha et al., 2020). There is a real need to streamline and simplify the way genetic data and associated patient phenotypes are collected and disseminated to the experimentalists who want to study these genes. This can be incredibly frustrating to the patient families who want to share their data and is complicated by the essential need for informed consent and rigorous ethical considerations. Currently, many model organism researchers find accessing these data challenging, slowing and even inhibiting progress.

Such access must be carefully balanced with the protection of patient data and there is no global consensus on what is considered ‘identifiable’ information. A model that facilitates the seamless translation and safe data sharing between the research and clinical space is, thus, a key requirement, whereby there is global equity of access for clinicians, researchers, patients and functional laboratories to collaborate and participate in research that will directly impact diagnostics and therapeutics. Efforts like the Center for Mendelian Genetics and the DECIPHER database provide a model for how this can be achieved at scale, and as patient groups grow and self-organise with help of foundations like COMBINEDbrain, there is the likelihood that these problems can be overcome. The *Xenopus* community is ready to meet patient and clinician needs.

It is clear from our experiences that there are more variants and genes of interest that need modelling than current resources allow. Model organisms are a core part of the solution to this major human health challenge. Within this space, the discussions throughout this article show that *Xenopus* has a key role, balancing its similarity with humans and broad experimental toolkit with its low cost and time effectiveness.
